# Supplementary material for: Modeling glioblastoma heterogeneity as a dynamic network of cell states
Source: Mol Syst Biol. 2021 Sep 16;17(9):e10105. doi: 10.15252/msb.202010105 (PMC8444284; doi:10.15252/msb.202010105)
Supplement: Supplementary file 6 — Source Data for Figure 5 [file MSB-17-e10105-s004.zip › Figure5A_sourcedata/GSEA_3071/hallmarks_stateVI.GseaPreranked.1623742557031/HALLMARK_E2F_TARGETS.html]

Details for gene set HALLMARK\_E2F\_TARGETS[GSEA]

|  || Dataset | state63071 |
| Phenotype | NoPhenotypeAvailable |
| Upregulated in class | na\_neg |
| GeneSet | HALLMARK\_E2F\_TARGETS |
| Enrichment Score (ES) | -0.34470192 |
| Normalized Enrichment Score (NES) | -1.6999187 |
| Nominal p-value | 0.0087241 |
| FDR q-value | 0.13025674 |
| FWER p-Value | 0.479 |
Table: GSEA Results Summary

  

Fig 1: Enrichment plot: HALLMARK\_E2F\_TARGETS      
 Profile of the Running ES Score & Positions of GeneSet Members on the Rank Ordered List

  

| PROBE | GENE SYMBOL | GENE\_TITLE | RANK IN GENE LIST | RANK METRIC SCORE | RUNNING ES | CORE ENRICHMENT || 1 | HMGA1 |  |  | 1 | 1.500 | 0.0299 | No |
| 2 | CDC20 |  |  | 15 | 1.073 | 0.0478 | No |
| 3 | MYC |  |  | 69 | 0.815 | 0.0493 | No |
| 4 | LYAR |  |  | 160 | 0.645 | 0.0368 | No |
| 5 | EIF2S1 |  |  | 287 | 0.547 | 0.0123 | No |
| 6 | PTTG1 |  |  | 344 | 0.514 | 0.0068 | No |
| 7 | NME1 |  |  | 405 | 0.487 | -0.0004 | No |
| 8 | AURKA |  |  | 572 | 0.432 | -0.0385 | No |
| 9 | PRDX4 |  |  | 647 | 0.412 | -0.0511 | No |
| 10 | HMMR |  |  | 681 | 0.404 | -0.0523 | No |
| 11 | RAN |  |  | 712 | 0.394 | -0.0529 | No |
| 12 | SNRPB |  |  | 817 | 0.374 | -0.0747 | No |
| 13 | TBRG4 |  |  | 884 | 0.363 | -0.0861 | No |
| 14 | DDX39A |  |  | 936 | 0.355 | -0.0933 | No |
| 15 | JPT1 |  |  | 965 | 0.350 | -0.0942 | No |
| 16 | CCNB2 |  |  | 967 | 0.350 | -0.0874 | No |
| 17 | UBE2S |  |  | 974 | 0.349 | -0.0821 | No |
| 18 | PA2G4 |  |  | 986 | 0.348 | -0.0782 | No |
| 19 | AK2 |  |  | 1032 | 0.339 | -0.0841 | No |
| 20 | POLE4 |  |  | 1222 | 0.312 | -0.1311 | No |
| 21 | POLD2 |  |  | 1265 | 0.308 | -0.1368 | No |
| 22 | NOP56 |  |  | 1531 | 0.276 | -0.2061 | No |
| 23 | DEPDC1 |  |  | 1545 | 0.275 | -0.2042 | No |
| 24 | SSRP1 |  |  | 1615 | 0.268 | -0.2183 | No |
| 25 | RAD50 |  |  | 1641 | 0.265 | -0.2201 | No |
| 26 | PLK1 |  |  | 1665 | 0.263 | -0.2213 | No |
| 27 | BIRC5 |  |  | 1667 | 0.263 | -0.2163 | No |
| 28 | NOLC1 |  |  | 1699 | 0.260 | -0.2198 | No |
| 29 | ILF3 |  |  | 1806 | -0.250 | -0.2447 | No |
| 30 | SLBP |  |  | 1819 | -0.252 | -0.2430 | No |
| 31 | MKI67 |  |  | 1825 | -0.253 | -0.2393 | No |
| 32 | RPA1 |  |  | 1849 | -0.256 | -0.2407 | No |
| 33 | STAG1 |  |  | 1895 | -0.263 | -0.2481 | No |
| 34 | AURKB |  |  | 1978 | -0.274 | -0.2657 | No |
| 35 | KIF22 |  |  | 2014 | -0.281 | -0.2700 | No |
| 36 | RFC2 |  |  | 2044 | -0.284 | -0.2724 | No |
| 37 | LUC7L3 |  |  | 2072 | -0.290 | -0.2742 | No |
| 38 | PRKDC |  |  | 2117 | -0.301 | -0.2806 | No |
| 39 | POLE |  |  | 2154 | -0.306 | -0.2846 | No |
| 40 | CDKN2C |  |  | 2197 | -0.313 | -0.2901 | No |
| 41 | TIMELESS |  |  | 2236 | -0.320 | -0.2944 | No |
| 42 | DCK |  |  | 2257 | -0.325 | -0.2935 | No |
| 43 | UNG |  |  | 2284 | -0.330 | -0.2942 | No |
| 44 | CENPM |  |  | 2287 | -0.330 | -0.2882 | No |
| 45 | LIG1 |  |  | 2290 | -0.331 | -0.2821 | No |
| 46 | ORC6 |  |  | 2343 | -0.342 | -0.2899 | No |
| 47 | DSCC1 |  |  | 2408 | -0.355 | -0.3008 | No |
| 48 | MCM5 |  |  | 2424 | -0.359 | -0.2978 | No |
| 49 | CBX5 |  |  | 2591 | -0.399 | -0.3367 | Yes |
| 50 | TCF19 |  |  | 2605 | -0.402 | -0.3323 | Yes |
| 51 | RBBP7 |  |  | 2609 | -0.403 | -0.3250 | Yes |
| 52 | SMC4 |  |  | 2634 | -0.408 | -0.3236 | Yes |
| 53 | STMN1 |  |  | 2646 | -0.411 | -0.3184 | Yes |
| 54 | RFC1 |  |  | 2690 | -0.421 | -0.3221 | Yes |
| 55 | ASF1B |  |  | 2704 | -0.427 | -0.3172 | Yes |
| 56 | MXD3 |  |  | 2717 | -0.430 | -0.3119 | Yes |
| 57 | RFC3 |  |  | 2765 | -0.446 | -0.3162 | Yes |
| 58 | MSH2 |  |  | 2786 | -0.452 | -0.3128 | Yes |
| 59 | DNMT1 |  |  | 2794 | -0.455 | -0.3056 | Yes |
| 60 | RAD21 |  |  | 2797 | -0.457 | -0.2970 | Yes |
| 61 | MCM7 |  |  | 2808 | -0.460 | -0.2905 | Yes |
| 62 | CTCF |  |  | 2820 | -0.462 | -0.2843 | Yes |
| 63 | MCM6 |  |  | 2834 | -0.468 | -0.2786 | Yes |
| 64 | MMS22L |  |  | 2867 | -0.484 | -0.2779 | Yes |
| 65 | UBE2T |  |  | 2868 | -0.484 | -0.2682 | Yes |
| 66 | GINS1 |  |  | 2877 | -0.486 | -0.2606 | Yes |
| 67 | SPC25 |  |  | 2896 | -0.493 | -0.2558 | Yes |
| 68 | BRCA2 |  |  | 2916 | -0.500 | -0.2511 | Yes |
| 69 | E2F8 |  |  | 2961 | -0.520 | -0.2531 | Yes |
| 70 | PSMC3IP |  |  | 2963 | -0.521 | -0.2429 | Yes |
| 71 | WEE1 |  |  | 2974 | -0.527 | -0.2351 | Yes |
| 72 | TMPO |  |  | 3022 | -0.552 | -0.2373 | Yes |
| 73 | NASP |  |  | 3049 | -0.563 | -0.2333 | Yes |
| 74 | MCM3 |  |  | 3064 | -0.570 | -0.2257 | Yes |
| 75 | SMC1A |  |  | 3067 | -0.571 | -0.2148 | Yes |
| 76 | LBR |  |  | 3068 | -0.572 | -0.2033 | Yes |
| 77 | DEK |  |  | 3098 | -0.583 | -0.1998 | Yes |
| 78 | PDS5B |  |  | 3177 | -0.641 | -0.2089 | Yes |
| 79 | MYBL2 |  |  | 3196 | -0.660 | -0.2007 | Yes |
| 80 | POLD3 |  |  | 3231 | -0.698 | -0.1962 | Yes |
| 81 | USP1 |  |  | 3233 | -0.699 | -0.1825 | Yes |
| 82 | LMNB1 |  |  | 3246 | -0.711 | -0.1715 | Yes |
| 83 | MCM4 |  |  | 3254 | -0.715 | -0.1591 | Yes |
| 84 | BARD1 |  |  | 3277 | -0.736 | -0.1505 | Yes |
| 85 | EZH2 |  |  | 3282 | -0.741 | -0.1368 | Yes |
| 86 | DUT |  |  | 3307 | -0.769 | -0.1281 | Yes |
| 87 | RRM2 |  |  | 3317 | -0.783 | -0.1149 | Yes |
| 88 | PSIP1 |  |  | 3328 | -0.794 | -0.1017 | Yes |
| 89 | BRCA1 |  |  | 3335 | -0.802 | -0.0873 | Yes |
| 90 | HELLS |  |  | 3361 | -0.826 | -0.0777 | Yes |
| 91 | RAD51AP1 |  |  | 3375 | -0.848 | -0.0643 | Yes |
| 92 | CCP110 |  |  | 3376 | -0.856 | -0.0471 | Yes |
| 93 | CDK1 |  |  | 3396 | -0.883 | -0.0347 | Yes |
| 94 | HMGB2 |  |  | 3414 | -0.922 | -0.0209 | Yes |
| 95 | PCNA |  |  | 3415 | -0.922 | -0.0024 | Yes |
| 96 | CDKN1B |  |  | 3426 | -0.951 | 0.0139 | Yes |
| 97 | TOP2A |  |  | 3457 | -1.020 | 0.0260 | Yes |
| 98 | ATAD2 |  |  | 3534 | -1.234 | 0.0294 | Yes |
Table: GSEA details [plain text format]

  

Fig 2: HALLMARK\_E2F\_TARGETS: Random ES distribution      
 Gene set null distribution of ES for **HALLMARK\_E2F\_TARGETS**
